# Rhizosphere Bacterial Community Characteristics over Different Years of Sugarcane Ratooning in Consecutive Monoculture

**DOI:** 10.1155/2019/4943150

**Published:** 2019-11-11

**Authors:** Xiaoning Gao, Zilin Wu, Rui Liu, Jiayun Wu, Qiaoying Zeng, Yongwen Qi

**Affiliations:** Guangdong Bioengineering Institute (Guangzhou Sugarcane Industry Research Institute), Guangdong Key Lab of Sugarcane Improvement and Biorefinery, Guangzhou, Guangdong 510316, China

## Abstract

To understand dynamic changes in rhizosphere microbial community in consecutive monoculture, Illumina MiSeq sequencing was performed to evaluate the V3-V4 region of 16S rRNA in the rhizosphere of newly planted and three-year ratooning sugarcane and to analyze the rhizosphere bacterial communities. A total of 126,581 and 119,914 valid sequences were obtained from newly planted and ratooning sugarcane and annotated with 4445 and 4620 operational taxonomic units (OTUs), respectively. Increased bacterial community abundance was found in the rhizosphere of ratooning sugarcane when compared with the newly planted sugarcane. The dominant bacterial taxa phyla were similar in both sugarcane groups. Proteobacteria accounted for more than 40% of the total bacterial community, followed by Acidobacteria and Actinobacteria. The abundance of Actinobacteria was higher in the newly planted sugarcane, whereas the abundance of Acidobacteria was higher in the ratooning sugarcane. Our study showed that *Sphingomonas*, *Bradyrhizobium*, *Bryobacter*, and *Gemmatimonas* were dominant genera. Moreover, the richness and diversity of the rhizosphere bacterial communities slightly increased and the abundance of beneficial microbes, such as *Bacillus*, *Pseudomonas*, and *Streptacidiphilus*, in ratooning sugarcane were more enriched. With the consecutive monoculture of sugarcane, the relative abundance of functional groups related to energy metabolism, glycan biosynthesis, metabolism, and transcription were overrepresented in ratooning sugarcane. These findings could provide the way for promoting the ratooning ability of sugarcane by improving the soil bacterial community.

## 1. Introduction

Sugarcane (*Saccharum officenarum* L.) is the most important sugar crop in China. Cane sugar accounts for approximately 90% of the total sugar produced in China, and it plays an important role in ensuring sugar safety and meeting the daily needs of people. At the same time, the sugar industry has become the primary source of income for many farmers in the region, enabling them to cast off poverty and set out on the road to prosperity [1]. Consecutive monoculture is a common method for sugarcane cultivation; it can increase efficiency and lower costs, while reducing the requirement for seeds and labor. However, there are challenges associated with the consecutive monoculture of ratooning sugarcane, including low yields, degradation of quality over time, and the increased presence of pests and diseases. Therefore, the development of new methods to extend the ratooning period has become a major focus in sugarcane production. The reasons behind challenges associated with consecutive monoculture are complex and are the result of long-term interactions between a plant, soil, and microbes [2]. Plant cultivation is a viable means to manipulate the composition and function of resident soil microbial communities. The microflora imbalance between the soil and roots leads to reduced microbial diversity and number of beneficial bacteria present and increases the number of pathogens and then hinders normal plant growth and development [3].

Hiltner first proposed the concept of rhizosphere in 1904, suggesting that the surface of the root system and the soil area directly affected by the root system be called the rhizosphere [4]. Metabolites present in the rhizosphere, such as organic acids and carbohydrates secreted by plant roots, provide favorable conditions for the growth and reproduction of microorganisms. Therefore, many soil microbes are active in the rhizosphere, making it the primary area for plant-soil-microbe interactions, material and energy exchanges, and signal communication [5].Therefore, many soil microbes are active in the rhizosphere, making this area the primary location for interactions among plants, microorganisms, and soil and their environment. The rhizosphere is an important interface for plant-soil-microbe interactions, material and energy exchange, and signal communication [5]. The number and variety of microorganisms within the rhizosphere are much higher than those outside it [5, 6]; rhizosphere microorganisms can affect plant growth and development, resistance to biotic and abiotic stresses (by altering osmotic potential), signal exchange rates, and enzyme activity during plant cell metabolism [7–10]. A large number of studies have shown that the microbial community structure in the rhizosphere is affected by many factors, such as the growth environment and the characteristics and developmental stages of the microbes present [11–13].

For the consecutive monoculture of sugarcane, it is of great importance to understand dynamic changes in rhizosphere microbial community in order to artificially intervene and improve ratooning ability. In this study, high-throughput sequencing was used to study the rhizosphere bacterial community of newly planted and three-year ratooning sugarcane. The characteristics of bacterial community in the rhizosphere of sugarcane were analyzed, and changes in the bacterial community of the newly planted and ratooning sugarcane were investigated. The results of our study could provide a new idea for overcoming the difficulties of ratooning sugarcane and promote sugarcane production.

## 2. Materials and Methods

### 2.1. Sampling Location and Samples Collection

Sampling was carried out in Dongsancun, Guandu Town, Wengyuan County, Shaoguan City, one of the major sugarcane planting areas in Guangdong Province (24°16′59″N, 113°56′24″E).

The sugarcane variety used in our study was Yuetang 08-196, which has good ratooning ability. In November 2018, rhizosphere soil samples of newly planted (planted in March, 2018) and three-year ratooning (planted in March, 2015) Yuetang 08-196 were collected. The five-point sampling method was used, and three plants were selected from each sample point. Litter and soil around the root were removed with a shovel. At a depth of 20–30 cm from the surface, the soil attached to the root was collected and placed in a 50 mL sterilized centrifuge tube. Samples from different sample points were mixed and numbered and brought back to the laboratory in an ice box. The samples were stored at –80°C for DNA extraction and high-throughput sequencing.

### 2.2. DNA Extraction

Three replicates of rhizosphere soil DNA from different treatments were extracted according to Fast DNA SPIN extraction kits (MP Biomedicals, Santa Ana, CA, USA). Afterwards, DNA concentration was measured by a spectrophotometer (NanoDrop 2000, ThermoScientific, USA) and the quality of DNA extracts was detected by 0.8% agarose gel electrophoresis.

### 2.3. PCR Amplification and Illumina MiSeq Sequencing

The bacterial community composition was assessed by sequencing the V3-V4 region of 16S rRNA gene using specific primers 338F (5′⁃ACTCCTACGGGAG -GCAGCA⁃3′) and 806R (5′⁃GGACTACHVGGGTWTCTAAT⁃3′). The PCR reactions were carried out in a 25 *μ*L reaction mixture containing 1.0 *μ*L each primer, 0.5 *μ*L dNTP, 10 *μ*L Buffer, 0.25 *μ*L Q5 High-Fidelity DNA Polymerase, 5 *μ*L High GC Enhancer, and 20 ng soil DNA template. The PCR conditions for bacteria were initiated at 98°C, 5 min (initial denaturation); 98°C, 10 s; 50°C, 30 s; 72°C, 30 s (for 25 cycles); 72°C, 5 min (final extension), and then at 4°C hold. Next, the PCR products were equally mixed. After full mixing, 2% agarose gel electrophoresis was used to detect the target bands, and QIAamp DNA Micro Kit (Qiagen, Valencia, CA, USA) was used to recover the target band products. Sequencing libraries were prepared by TruSeq Nano DNA LT Library Prep Kit (Illumina, USA). The constructed libraries were checked by Agilent Bioanalyzer, and the qualified libraries were sequenced by Illumina MiSeq platform (Shanghai Personal Biotechnology Co., Ltd, China).

### 2.4. Data Analysis

Paired-end sequencing of community DNA fragments was carried out on the Illumina MiSeq platform. The raw sequencing data were screened for quality and merged by FLASH software. The merged reads were identified and assigned to the corresponding samples to obtain the valid sequences of each sample. QIIME pipeline was used to identify the questioning sequence, to check and filter the chimeric reads, and to count the high-quality sequence numbers. The UCLUST sequence-matching tool was used to merge the high-quality sequences and assign operational taxonomic units (OTUs) at 97% similarity. The number of OTUs shared by each sample was calculated using R software, and the proportion of common and unique OTUs for each sample was presented by the Venn diagram [14, 15]. Mothur software and the statistical algorithm of Metastats [16] were used to perform a pairwise comparison of the differences in sequence quantity (i.e., absolute abundance) between the samples (groups) of each taxon at the phylum and genus levels. The UniFrac distance of different groups can be presented in boxplots. Combined with the statistical results, boxplots are helpful to comprehensively describe the differences between the structures of microbial communities within and between groups, and to analyze the taxonomic composition of the community at the phylum and genus levels, thus showing the main distribution characteristics of the community samples. The top 50 most abundant genera were clustered and analyzed to plot the heatmap. A linear discriminant analysis (LDA) effect size (LEfSe) was performed to evaluate the bacterial taxa differentially represented between the different treatments [17]. The PICRUSt method was used to compare the 16S rRNA sequencing data with the KEGG database and to compare the abundance differences of metabolic pathway.

## 3. Results

### 3.1. Sequencing Results and Microbial Diversity Analysis

**Figure 1:**
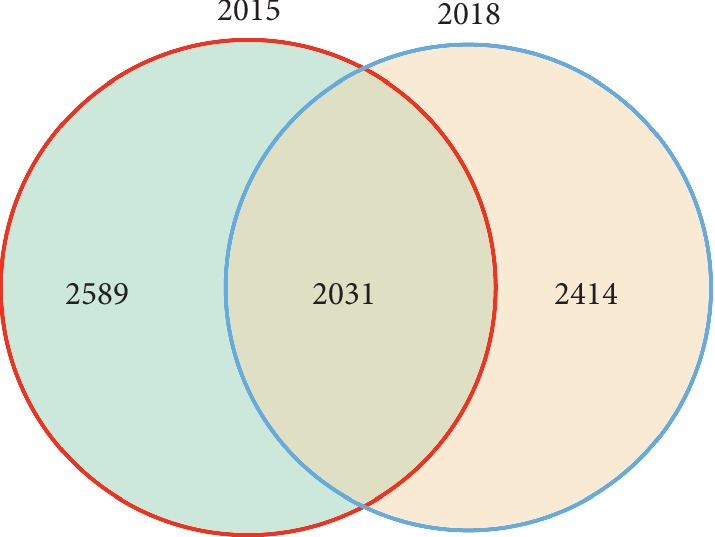
Venn diagram of OTUs between the three-year ratooning sugarcane (2015) and the newly planted sugarcane (2018).

A total of 126,581 and 119,914 valid sequences were obtained from newly planted and ratooning sugarcane valid sequences from the raw data after splicing ([Supplementary-material supplementary-material-1]). Sequences were clustered by OTUs at 97% similarity, and the abundance of different OTUs in all samples was obtained. The Venn diagram (Figure 1) shows that there were 4,445 OTUs in rhizosphere soil samples of newly planted sugarcane and 4,620 OTUs in samples from three-year ratooning sugarcane, which indicated that the rhizosphere bacterial community of ratooning sugarcane was richer than that of the newly planted sugarcane.

The bacterial richness (Chao1 and ACE) and community diversity (Shannon index) were estimated. There were no significant differences of bacterial richness and community diversity in the rhizosphere soil of the three-year ratooning sugarcane compared with the newly planted sugarcane (Table 1).

### 3.2. Microbial Community Structure

**Figure 2:**
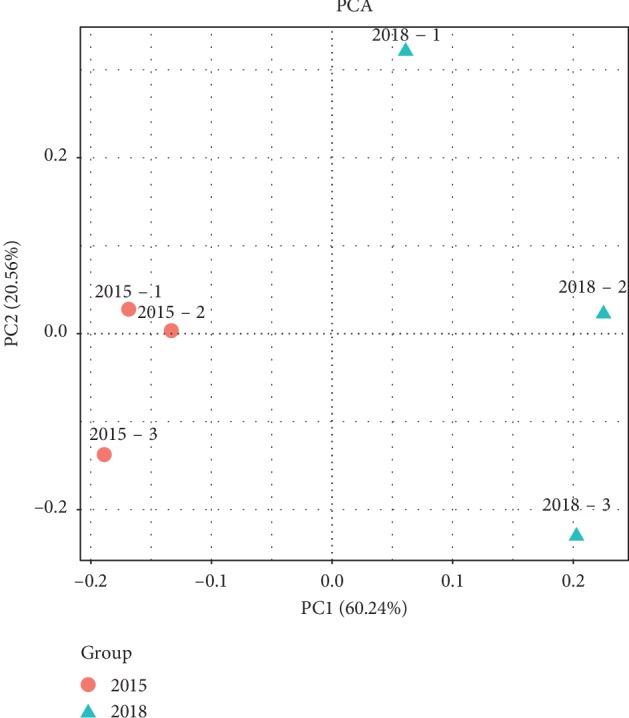
Principal component analysis based on the distance matrix calculated using the Bray–Gurtis algorithm for soil samples collected from the rhizosphere of the three-year ratooning sugarcane (2015) and the newly planted sugarcane (2018).

A principal coordinate analysis (PCoA) based on the Bray–Curtis algorithm clearly revealed that the soil microbial community structures varied among treatments (Figure 2). Two treatments were clearly separated from each other. Of the total variance in the dataset, the first two principal components together explained 80.80% of the total bacterial communities. In addition, the first principal component (PC1) was the most important, accounting for 60.24% of the total variation of the bacterial communities.

**Figure 3:**
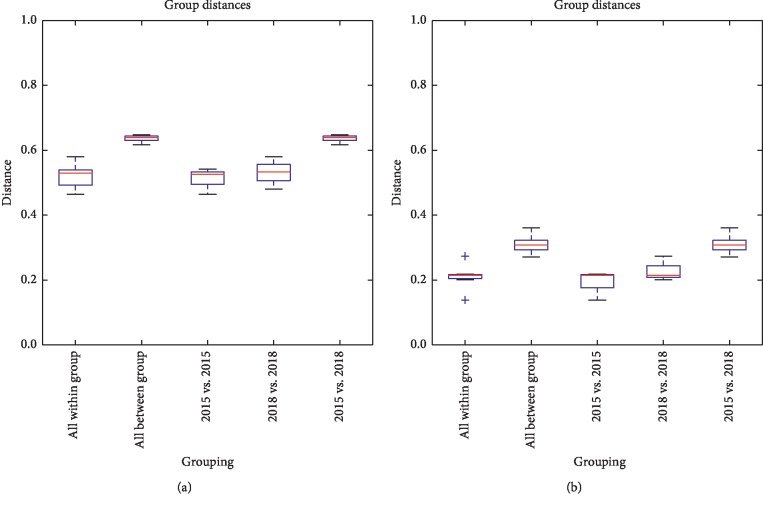
NMDS plot comparison based on Unweighted UniFrac distance (a) and Weighted UniFrac distance (b).

NMDS were obtained to compare and analyze the differences between and within groups, which can directly reflect the differences in bacterial community structure. The structure of rhizosphere bacterial communities pertaining to newly planted sugarcane and three-year ratooning sugarcane were compared between and within groups.  Figure 3 shows that there was a significant difference between sugarcane groups and that this difference was significantly higher than the differences within groups. These results suggested that there were significant differences in rhizosphere bacterial community between the newly planted sugarcane and the three-year ratooning sugarcane.

### 3.3. Rhizosphere Bacterial Taxonomic Composition

**Figure 4:**
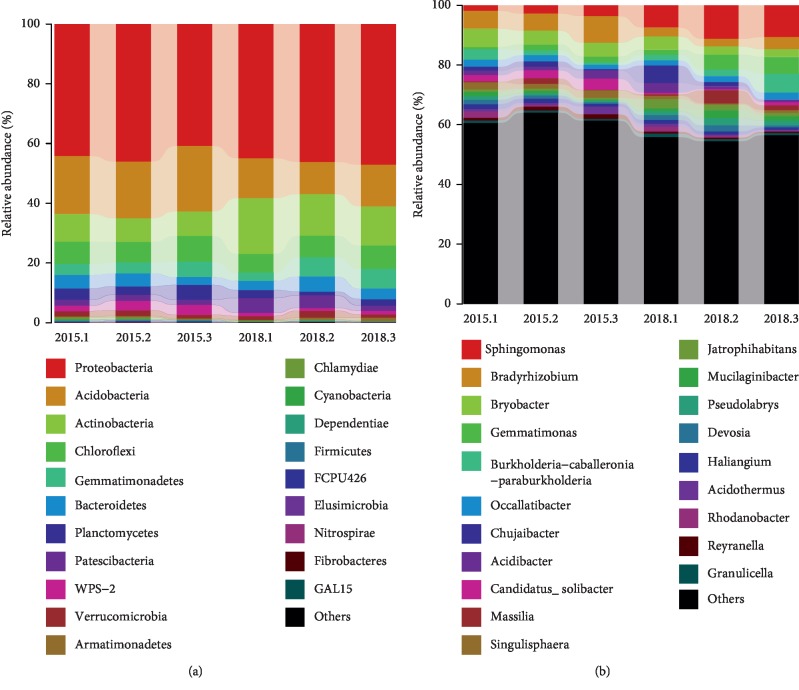
The relative abundance of the top 20 bacterial phyla (a) and genera (b) for rhizosphere from the three-year ratooning sugarcane (2015) and the newly planted sugarcane (2018).

Based on the analysis of the top 20 most abundant bacterial phyla, the dominant phyla in the two sugarcane groups included Proteobacteria, Acidobacteria, Actinobacteria, Chloroflexi, Gemmatimonadetes, Bacteroidetes, Planctomycetes, and Patescibacteria (Figure 4(a)). Proteobacteria was the most dominant phylum, accounting for more than 40% of the total number of species present, followed by Acidobacteria and Actinobacteria. In the same habitat, the proportion of each phylum in the sugarcane rhizosphere varied between different ratooning years. In the rhizosphere of three-year ratooning sugarcane, Acidobacteria accounted for a higher proportion, while Actinobacteria accounted for a lower proportion when compared to the rhizosphere of the newly planted sugarcane.

At the genus level, the dominant genera of the two sugarcane groups included *Sphingomonas, Bradyrhizobium, Bryobacter, Gemmatimonas, Burkholderia, Occallatibacter*, and *Chujaibacte* (Figure 4(b)). *Sphingomonas*, *Bradyrhizobium*, *Bryobacter*, and *Gemmatimonas* were the most dominant genera. However, the proportion of dominant bacterial genera in the rhizospheres of newly planted sugarcane and ratooning sugarcane were significantly different. In the rhizosphere of three-year ratooning sugarcane, the proportion of *Sphingomonas* decreased from 9.7% (present in rhizosphere of newly planted sugarcane) to 2.7%, whereas the proportion of *Bradyrhizobium* and *Bryobacter* increased with the extension of ratooning.

**Figure 5:**
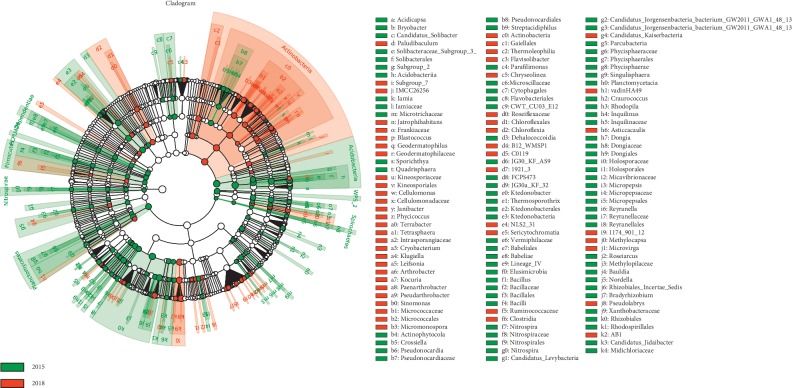
Comparison of microbial variations based on the LEfSe analysis in the three-year ratooning sugarcane (2015) and the newly planted sugarcane (2018). Differences are represented by the color of the taxa (green indicating the three-year ratooning sugarcane and red indicating the newly planted sugarcane).

**Figure 6:**
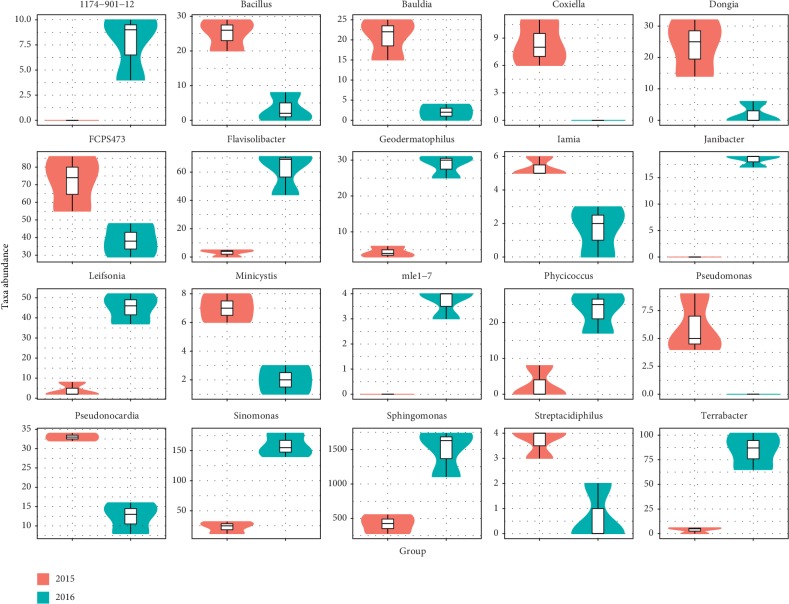
The abundance distributions of the top 20 genera between the rhizospheres of the three-year ratooning sugarcane (2015) and the newly planted sugarcane (2018).

Bacterial taxa with significantly different abundances were detected by using LEfSe between the rhizosphere of three-year ratooning sugarcane and newly planted sugarcane (Figure 5). The most differentially abundant bacterial taxa in the newly planted sugarcane rhizosphere soils belong to the Actinobacteria phylum, whereas the phylum Acidobacteria, Planctomycetes, Nitrospirae, and Firmicutes were more abundant in the three-year ratooning sugarcane rhizosphere soils (Figure 5). Moreover, at the genus level, 69 genera showed significant differences between the rhizospheres of the newly planted and ratooning sugarcane. The abundance distributions of the top 20 genera with the greatest significant differences were analyzed (Figure 6). The genera *Bacillus*, *Bauldia*, *Coxiella*, *Dongia*, *Iamia*, *Minicystis*, *Pseudomonas*, *Pseudonomia*, and *Streptacidiphilus* were overrepresented in the rhizosphere bacterial community of ratooning sugarcane compared to newly planted sugarcane.

### 3.4. Rhizosphere Microbial Community Function

**Figure 7:**
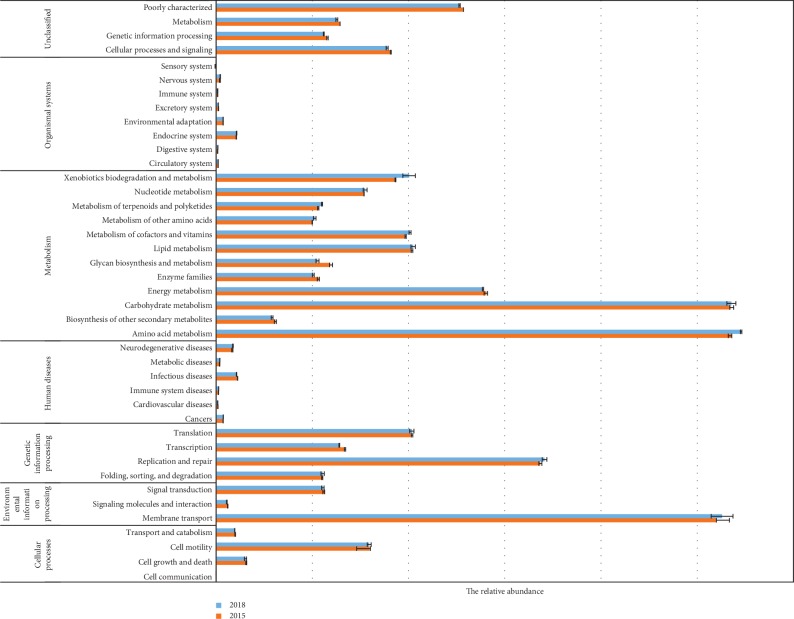
The relative abundance of microbial community functions between the rhizospheres of the newly planted sugarcane (2018) and the three-year ratooning sugarcane (2015).

Predictions of the Kyoto Encyclopedia of Genes and Genomes (KEGG) orthologs (KOs) and pathways were performed on the 16S rRNA gene soil bacterial composition data by using the PICRUSt (Figure 7). The relative abundance pathways related to metabolism, genetic information processing, environmental information processing, and cellular processes were higher in the two treatments. Compared with the newly planted sugarcane, the relative abundance of five pathways, including glycan biosynthesis and metabolism, enzyme families, energy metabolism, biosynthesis of other secondary metabolites, and transcription was significantly higher in the ratooning sugarcane.

## 4. Discussion

In our study, Illumina MiSeq high-throughput sequencing was used to analyze the differences between the rhizospheres bacterial communities of the newly planted and three-year ratooning sugarcane. Previous studies have found that plants can interact with soil and microorganisms [7, 18, 19]. The results from our study showed that there were some similarities in bacterial community composition between the newly planted and ratooning sugarcane rhizospheres. Proteobacteria, Acidobacteria, and Actinobacteria were the main phyla in rhizosphere bacterial communities. This finding roughly corresponded with those of previous articles that investigated agricultural or other type soils in which these phyla of the sequences that were examined using deep 16S rRNA pyrosequencing [20–22]. Proteobacteria is a relatively abundant phylum that is commonly found in soil [23], and its relative abundance is much higher than those of other phyla in this study. Acidobacteria is a phylum that widely exists in the plant rhizosphere, can degrade polysaccharides, and may play an important role in carbon cycling [24]. Actinobacteria in rhizosphere is likely to be determined by several different selective factors that influence the growth and the size of different Actinobacterial structures [25]. Actinobacteria phylum was consistently associated with disease suppression, since they have higher abundances in many disease-suppressive soils than in disease-conducive soils [26–29].

We found that the difference between the bacterial communities of newly planted and ratooning sugarcane rhizospheres were most apparent in Acidobacteria, Actinobacteria, Firmicutes, and Spirochetes ([Supplementary-material supplementary-material-1]), among which, the proportion of Actinobacteria in the rhizosphere bacterial community of ratooning sugarcane was 41.4% lower than that of newly planted sugarcane. Actinobacteria are believed to play an important role in promoting plant growth in rhizosphere soils and to regulate the biological interactions between plants, pathogens, and the microenvironment [30, 31]. In this study, the relative abundance of Actinobacteria dramatically decreased with the extension of sugarcane ratooning. Therefore, further research is needed to confirm whether Actinobacteria communities play a similar role in sugarcane rhizospheres.

The abundance of soil microbes reflected the diversity of soil microbial community. The dominant genera detected in this study differed greatly between the rhizospheres of newly planted and ratooning sugarcane ([Supplementary-material supplementary-material-1]). The difference in the distribution of dominant bacteria in rhizosphere soil indicated that sugarcane engages in complex interactions with rhizosphere bacteria during the process of ratooning. Rhizosphere microbial diversity plays a key role in the promotion of plant growth and health [5]. The results of this study showed that *Sphingomonas, Bradyrhizobium*, and *Gemmatimonas* were the dominant genera in the sugarcane rhizosphere bacterial community. Previous studies have shown that *Sphingomonas* has a strong ability to degrade environmental pollutants and can promote the absorption and growth of plants. It has also been shown that *Sphingomonas* is the primary antimicrobial agent in soil communities and that this group has an inhibitory effect on plant pathogenic fungi [32–36]. *Bradyrhizobium* is a common soil microorganism that can establish mutually beneficial symbiotic relationships with plant roots and fix nitrogen [37–39]. *Gemmatimonas* is a newly established genus that exists widely in aquatic and terrestrial habitats and has been found to be highly abundant in the rhizospheres of healthy plants [22, 40]. However, in sugarcane rhizosphere, the effects of *Sphingomonas, Bradyrhizobium*, and *Gemmatimonas* on the sugarcane ratooning ability, nutrient uptake, and the roots growth need to be further studied.

Chen et al. [41] and Wang et al. [42] showed that some beneficial microbial groups such as the plant growth-promoting rhizobacteria (PGPR, mainly *Bacillus* and *Paenibacillus*) increased significantly in *Achyranthes bidentate* with the extension of consecutive monoculture. The results of Zhu et al. also showed that the richness and diversity of the soil bacterial community increased slightly after a long-term consecutive soybean monoculture and that the proportion of *Bradyrhizobium* and *Nitrospira*, which can promote the nutrient uptake and the growth of plants [43]. In our study, we also found that some beneficial microbial groups such as *Bacillus*, *Pseudomonas, Streptacidiphilus*, and *Bradyrhizobium* increased significantly in the rhizosphere bacterial communities of three-year ratooning sugarcane when compared with the rhizospheres of newly planted sugarcane. This indicates that beneficial microbial groups can be enriched in the rhizosphere and may play an active role in the ratooning of sugarcane. These results also provide the way for improving the ratooning ability of sugarcane through the modification of soil microbial community.

Understanding the function of the microbiomes is key for understanding their interrelationships with the environment. The relatively recently developed PICRUSt program has proved to be effective at obtaining functional predictions from 16S rRNA taxonomic data [44]. Comparisons of the same functional pathways between the rhizosphere bacteria of the ratooning sugarcane and the newly planted sugarcane showed that their abundance between groups was significantly different. With the consecutive monoculture of sugarcane, the relative abundance related to energy metabolism, glycan biosynthesis and metabolism, and transcription was greater in the rhizosphere of ratooning sugarcane, indicating that sugarcane require more energy for metabolism and environmental adaptation during the process of ratooning.

In this study, sugarcane rhizosphere bacterial community was analyzed by high-throughput sequencing. Based on the current findings, we will further study composition and variation of microbial communities in the rhizospheres and bodies of sugarcane with different ratooning abilities and at different developmental stages. These studies will help to understand the interactions between microorganisms and sugarcane in a more comprehensive manner and, thus, lay the foundation for overcoming challenges associated with ratooning and promote healthy sugarcane production.

## 5. Conclusion

In this study, we firstly compare the rhizosphere bacterial community characteristics over different years of sugarcane rationing in consecutive monoculture by Illumina MiSeq sequencing. There were differences in the dominant rhizosphere bacterial taxonomic composition and community functions between the ratooning and newly planted sugarcane. The richness of the rhizosphere bacterial communities and the abundance of beneficial microbes, such as *Bacillus, Pseudomonas*, and *Streptacidiphilus*, increased in ratooning sugarcane. Thus, these findings could provide the way for promoting the ratooning ability of sugarcane by improving the soil bacterial community.

## Figures and Tables

**Table 1:** Calculations of Chao1, ACE, and Shannon indices for treatments of three-year ratooning sugarcane (2015) and the newly planted sugarcane (2018).

Treatments	Chao1	ACE	Shannon
2015	2847.81 ± 560.84	2922.89 ± 659.04	9.81 ± 0.10
2018	2779.16 ± 545.64	2865.55 ± 572.70	9.73 ± 0.17

Values indicate the means followed by standard error of the mean.

## Data Availability

The data used to support the findings of this study are available from the corresponding author upon request.

## Supplementary Materials

Supplementary Materials Table S1: good quality sequences that were used for further analysis after basic quality control for treatments of three-year ratooning sugarcane (2015) and the newly planted sugarcane (2018). Figure S1: the abundance distributions of the seven phyla between the rhizospheres of the newly planted sugarcane (2018) and the three-year ratooning sugarcane (2015). Figure S2: heatmap of the top 50 classified bacterial genera of the rhizosphere of the newly planted sugarcane (2018) and the three-year ratooning sugarcane (2015) and Metagenome at level 3.
