# Supplementary material for: Rhizosphere Bacterial Community Characteristics over Different Years of Sugarcane Ratooning in Consecutive Monoculture
Source: Biomed Res Int. 2019 Nov 11;2019:4943150. doi: 10.1155/2019/4943150 (PMC6878781; doi:10.1155/2019/4943150)
Supplement: Supplementary Materials — Table S1: good quality sequences that were used for further analysis after basic quality control for treatments of three-year ratooning sugarcane (2015) and the newly planted sugarcane (2018). Figure S1: the abundance distributions of the seven phyla between the rhizospheres of the newly planted sugarcane (2018) and the three-year ratooning sugarcane (2015). Figure S2: heatmap of the top 50 classified bacterial genera of the rhizosphere of the newly planted sugarcane (2018) and the three-year ratooning sugarcane (2015) and Metagenome at level 3. [file 4943150.f1.zip › 4943150.f1/Supplement table and figure.doc]

TABLE S1. Good quality sequences that were used to further analysis after basic quality control for treatments of three-year ratooning sugarcane (2015) and the newly planted sugarcane (2018).

| Treatments | Good quality sequences |
| --- | --- |
| 2015-1 | 42752 |
| 2015-2 | 39761 |
| 2015-3 | 37401 |
| 2018-1 | 39575 |
| 2018-2 | 46359 |
| 2018-3 | 40647 |
| Total | 246495 |

FIGURE S1: The abundance distributions of the seven phyla between the rhizospheres of the newly planted sugarcane (2018) and the three-year ratooning sugarcane (2015).

FIGURE S2 Heatmap of the top 50 classified bacterial genera of the rhizosphere of the newly planted sugarcane (2018) and the three-year ratooning sugarcane (2015).
